# Periodontal Diseases: Bug Induced, Host Promoted

**DOI:** 10.1371/journal.ppat.1004952

**Published:** 2015-07-30

**Authors:** Shariq A. Khan, Eric F. Kong, Timothy F. Meiller, Mary Ann Jabra-Rizk

**Affiliations:** 1 Department of Oncology and Diagnostic Sciences, Dental School, University of Maryland, Baltimore, Maryland, United States of America; 2 Graduate Program in Life Sciences, Molecular Microbiology and Immunology Program, University of Maryland, Baltimore, Maryland, United States of America; 3 Department of Microbiology and Immunology, School of Medicine, University of Maryland, Baltimore, Maryland, United States of America; Duke University Medical Center, UNITED STATES

## Introduction

Periodontal disease (PD) is one of the most ubiquitous diseases of mankind, considered to be the second most common dental disease worldwide, after dental decay. This chronic condition is characterized by a complex group of inflammatory diseases affecting the periodontium, or the tissues that surround and support the teeth. If left untreated, this condition can lead to progressive loss of the alveolar bone around the teeth and subsequent loss of teeth. In fact, PD remains the most common cause of tooth loss in the world today; in the United States, it has a prevalence of 30%–50% of the population and can affect up to 90% of the population worldwide [1]. Like other conditions intimately related to access to hygiene and basic medical monitoring, periodontitis tends to be more common in economically disadvantaged populations. Interestingly, in addition to humans, periodontitis is the most common disease found in dogs affecting more than 80% of dogs aged three years or older. The complex nature of PD involving interactions between microbial and host factors has made this disease entity difficult to study. Here we provide a brief account of our current concepts of PD, its etiology, and the relevant clinical implications of this prevalent inflammatory disease.

## Risk Factors and Diagnosis

Numerous modifying factors are associated with PD. Most notable is lack of oral hygiene with inadequate plaque biofilm control. However, smoking is also strongly associated with periodontitis, as studies have shown that tobacco negatively affects periodontal tissues [2]. Furthermore, genetic susceptibility, poor nutrition, and age are also other important factors related to the incidence of periodontal destruction, with the highest rate occurring between 50 and 60 years of age. Significantly, individuals with HIV infection and AIDS are prone to aggressive forms of PD, particularly necrotizing ulcerative periodontitis (NUP), which is a sign of severe suppression of the immune system.

**Fig 1:**
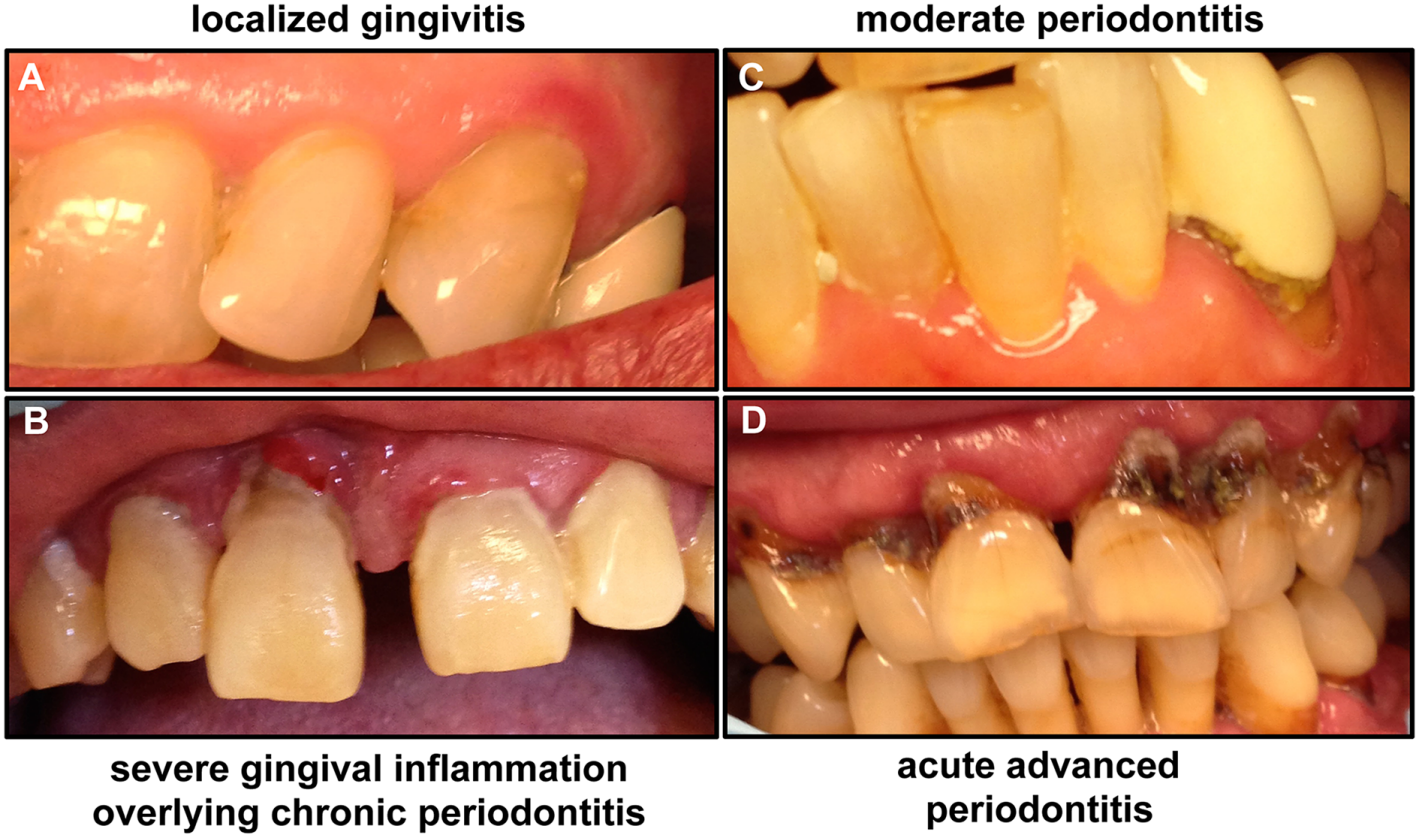
Images of patients demonstrating the clinical progression of periodontal disease from gingivitis to advanced periodontitis. **(A)** If not treated, mild gingivitis leads to **(B)** severe gingival inflammation and **(C)** periodontitis characterized by separation of gingival tissue from the tooth, followed by **(D)** severe periodontitis, which eventually results in loss of teeth. Images taken are of patients attending the Oral Medicine Clinic at the University of Maryland School of Dentistry.

**Fig 2:**
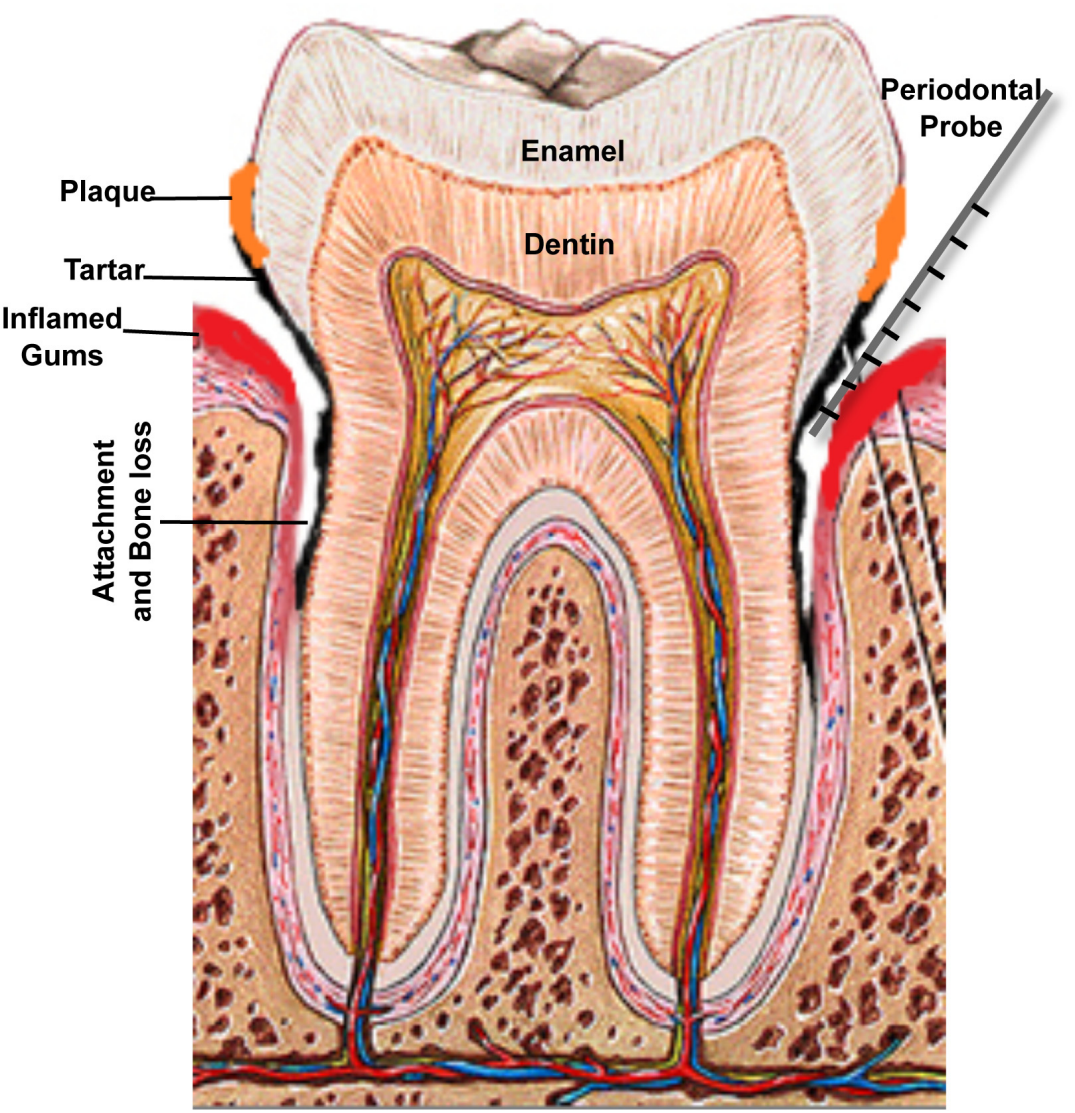
A schematic of the human tooth illustrating the process of periodontal disease development. Accumulation of dental plaque leads to formation of tartar and inflammation of gingival tissue. Separation of the gum from the tooth creates a periodontal pocket and loss of bone support. Measuring disease progression is carried out by measuring pocket depth around the teeth, using a periodontal probe; pockets greater than 3 mm in depth are considered to be unhealthy, and 7 mm or deeper pockets indicate significant loss of attachment and carry risk of eventual tooth loss.

A diagnosis of periodontitis is established by clinical examination of the soft gum tissues and by radiographic examination to determine the amount of bone loss around the teeth. Periodontal diseases can be broadly classified as aggressive or chronic, beginning with gingivitis (Fig 1). Gingivitis itself does not affect the underlying supporting structures of the teeth and is reversible; however, in some people, gingivitis progresses, resulting in destruction of the gingival fibers and connective tissue. This process causes the gum tissues to separate from the tooth, creating a periodontal pocket and loss of bone support. Therefore, chronic periodontitis is characterized by gum swelling and bleeding on probing, gingival recession, and deep pockets between the teeth and the gums that lead to loosening of the teeth and, ultimately, tooth loss [1]. Monitoring disease progression is carried out by measuring pocket depth and bleeding indices using a device called a periodontal probe placed into the space between the gums and the teeth and slipped below the gumline (Fig 2). Pockets greater than 3 mm in depth are considered to be unhealthy, and bleeding on probing is considered to be a sign of active disease; if patients have 7 mm or deeper pockets around their teeth, then they would likely risk eventual tooth loss.

## Etiology: The Bug Aspect

It is generally agreed that the primary etiology of gingivitis is adherence and growth of microbial species on the surface of teeth, forming dental plaque [3]. In fact, microorganisms were first considered as possible etiological agents of periodontitis in the late 1800s [4]. If accumulation of microbial biofilm at the gumline is left undisturbed, plaque calcifies to form calculus or tartar, which then leads to chronic inflammation of the periodontal tissues (Fig 2). The microbiology of periodontitis is complex, and therefore, despite considerable recent attention to the composition of the human microbiome, the mechanisms underlying the intricate microbial interactions that lead to inflammatory diseases such as periodontitis remain poorly defined [5,6]. Most simplistically, PD has been characterized as a microbial-shift disease owing to shift in the subgingival microbial communities that colonize the periodontal pockets from a predominantly Gram-positive aerobic bacteria, to a dominance of Gram-negative anaerobes during the transition from periodontal health to PD [4]. Therefore, periodontitis is essentially induced by a dysbiotic microbiota. This concept of periodontal pathogenesis was recently termed “polymicrobial synergy and dysbiosis,” or the PSD, model by Hajishengallis et al. [7].

It is probable that commensal bacteria induce a protective response that prevents the host from developing disease. However, several bacterial species found in plaque use various mechanisms to interfere with host defense mechanisms and studies have revealed that many oral bacteria can elicit host responses with varying potencies [4]. Extensive sequencing studies unraveling the oral microbiome have identified consortia of different bacterial species associated with severe chronic periodontitis [8]. Among the Gram-negative bacteria considered the main periodontal pathogens are *Tannerella forsythia*, *Prevotella intermedia*, *Fusobacterium nucleatum*, and *Eubacterium* sp., with *Actinomyces actinomycetemcomitans*, *Campylobacter rectus*, and *Eikenella corrodens* also likely playing a role in chronic periodontitis. However, the most notable are the so-called “red-complex” bacteria: *Porphyromonas gingivalis*, *Tannerella forsythia*, and *Treponema denticola*.

*Porphyromonas gingivalis* specifically has long been identified as the “keystone pathogen” as this species is detected infrequently and in low numbers in health, and in greater frequency in destructive forms of the disease [9]. This pathogen has an impressive armamentarium of virulence factors, including fimbrae, degradative enzymes, and exopolysaccharide capsule. However, most significant are a group of secreted cysteine proteases with proteolytic activity known as gingipains, which cleave host proteins and are therefore associated with tissue damage and immune disruption. The critical role of gingipains in *P*. *gingivalis*–mediated disease pathogenesis was clearly evidenced by the attenuated virulence exhibited by gingipain knockout *P*. *gingivalis* [10]. Of more significance, however, is that at very low colonization levels, *P*. *gingivalis* is able to trigger changes to the amount and composition of the oral commensal microbiota, thereby facilitating a dysbiotic shift in community. Furthermore, *P*. *gingivalis* also helps suppress the immune system in a way that creates a hospitable environment for the other bacteria. Using a murine periodontal model, *P*. *gingivalis*, even in low numbers, was shown to orchestrate inflammatory periodontitis through interactions with oral commensal microbiota and the complement system [10]. These findings are of significance as they demonstrate that a single, low-abundance species can disrupt host-microbial homeostasis to cause inflammatory disease. However, the definition of the relationship between oral microbial consortia and disease has been precluded by our inability to study complex microbial interactions within the host. Therefore, the contributions of the different microbial communities associated with either health or disease and the mechanisms that maintain the stability of or induce changes in the microbial composition remain unclear.

## The Host Perspective

**Fig 3:**
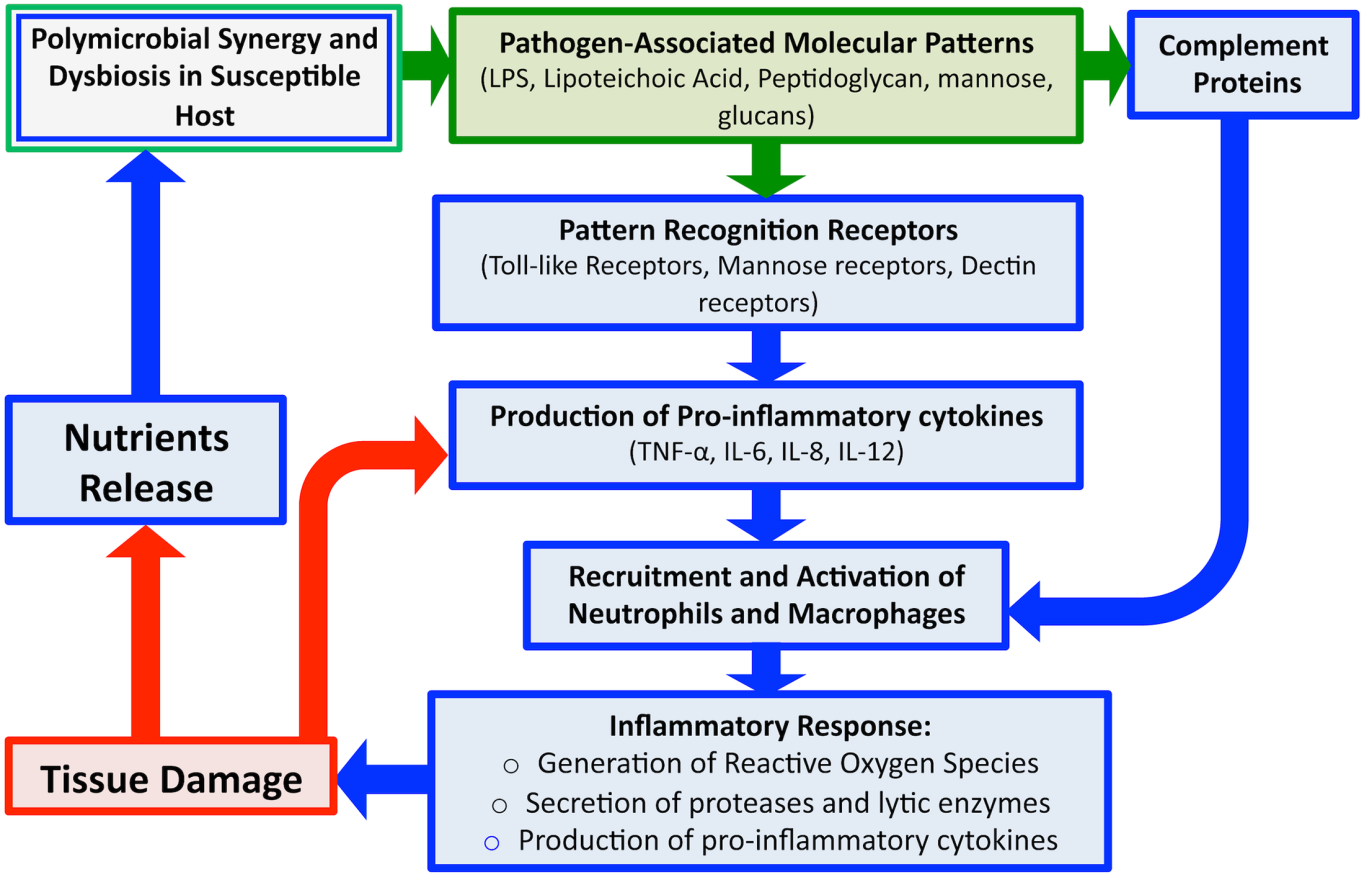
An overview schematic of the microbial and host-associated pathologies of periodontal disease. Disease is initiated via dysregulation between the polymicrobial microbiota and the host immune barriers. Detection of microbial PAMPs by host immune PRRs evokes rapid induction of immune response and production of pro-inflammatory cytokines, which along with certain components of complement, results in recruitment of host immune cells. Degranulation of polymorphonuclear cells (PMNs) releases proteases and other lytic enzymes; localization of these inflammatory reactions results in tissue breakdown, providing nutrients for bacteria, in turn, inducing a feed-forward loop of disease progression. Therefore, although the initial trigger of gingival inflammation is microbial (green), the damage to tissue is largely host associated (blue).

The recognition that the host contributes to the pathology of periodontitis was a major conceptual advance (Fig 3) [11]. Pattern recognition receptors (PRRs) on host immune cells detect key molecular patterns on microbial organisms (pathogen-associated molecular patterns, or PAMPs) such as lipopolysaccharides (LPS). The proper engagement of PAMPs evokes rapid induction of immune response, which in turn facilitates the activation and recruitment of key immune components via the production of cytokines and chemokines. Detection of these microbial products allows for the maintenance and surveillance of microbial colonization, and therefore, host oral microbiota maintains a homeostatic balance with the host immune system [12].

Similarly, antimicrobial peptides (AMPs) secreted by the oral mucosa and complement proteins of the host immune system also act to control microbial colonization and limit spread. However, certain components of complement can also act to recruit polymorphonuclear cells (PMNs) to enhance the inflammatory response. Since tissue PMNs and resident macrophages respond rapidly to breaches in the tissue epithelium, breaching of the mucosal barrier is a highly immunogenic event [13,14]. Although induced immune responses contribute to maintaining homeostasis with commensal organisms, when these inflammatory reactions are localized in the tissue, they result in damage, as is the case with periodontitis, in which initial periodontal lesions trigger recruitment of PMNs to the damaged areas [13,14]. Additionally, degranulation of PMNs results in release of proteases and other lytic enzymes, which can help to eliminate the microbial burden, but also results in additional local damage to host tissue through the destruction of connective tissue fibers. In addition to enzymes, PMNs activation also results in production of pro-inflammatory cytokines such as interleukin (IL)-1 and tumor necrosis factor (TNF), which further enhance inflammation [8]. In normal infections, engagement of the adaptive arm of the immune system is normally sufficient to clear the pathogen, but in the oral cavity, the continuous microbial signals emanating from biofilm plaque prevent the immune response from resolving the infection, and inflammation persists, causing damage to the gingiva [8,11]. In addition to tissue damage, several osteoclast-related mediators target the alveolar bone, causing bone resorption and demineralization, which results in bone destruction. Therefore, although the initial trigger of gingival inflammation is microbial, the damage to host tissue is largely mediated by the immune response. However, how bacterial modulation of host cytokine expression and host immune repertoires may lead to destructive PD is yet to be fully elucidated.

## Therapeutic Interventions

The primary goal of periodontal treatment is to restore the homeostatic relationship between periodontal tissue and its polymicrobial dental-plaque community. Therefore, prevention and treatment are primarily aimed at controlling the bacterial biofilm and other risk factors, arresting progressive disease, and restoring lost tooth support. The most widely used treatment is physical removal of the plaque by scaling [15]. Therefore, establishing proper oral hygiene by practicing daily measures such as brushing, flossing, using an antiseptic mouthwash, and regular dental check-ups is the cornerstone for successful prevention and treatment of periodontal diseases. Gingivitis, which is the mildest form of the disease, is readily reversible by simple, effective oral hygiene. However, if periodontal disease develops, the typical initial nonsurgical treatment is a procedure called scaling and debridement to mechanically remove microbial plaque and calculus, which is done using a powered ultrasonic or sonic scaler. Adjunctive antimicrobials such as chlorhexidine and systemic antibiotic treatment such as amoxicillin or metronidazole are sometimes used in addition to debridement-based treatments, as well as extended-release antibiotics such as doxycycline [15]. If nonsurgical therapy is unsuccessful in managing disease activity, periodontal surgery may be needed to stop progressive bone loss and regenerate lost bone where possible; this may involve flap surgery, soft tissue and bone grafts, or guided tissue regeneration [16]. Recently, however, studies have increasingly focused on using laser systems as an adjunct in periodontal therapy. One such system is the use of photodynamic therapy, which proved to be very effective in reducing bacteria, promoting healing of gums, and helping regain lost attachment [17]. Other therapeutic interventions include vaccine strategies targeting key bacteria; specifically, immunization directed against *P*. *gingivalis* has shown efficacy in preclinical studies [4]. Therefore, identification and targeting of similar low-abundance pathogens with community-wide impact may be important for treating inflammatory diseases of polymicrobial etiology. However, recently important strides have been made in a pioneering study by Maekawa et al. [18] identifying a central role for the C3 component of the complement system in the cascade that triggers and maintains inflammation. Building on these findings, a drug that blocks C3 was developed, which was shown to be potent in inhibiting inflammatory processes in nonhuman primates, strongly supporting the feasibility of C3-targeted intervention for the treatment of human periodontitis. These findings were also supported by studies demonstrating that C3-deficient mice are protected from periodontitis. An additional advantage of anti-inflammatory treatments is that blocking inflammation deprives bacteria of the nutrients they derive from inflammatory breakdown of tissue.

## Systemic Effects of Periodontitis

Periodontitis has been linked to increased inflammation in the body, such as indicated by raised levels of C-reactive protein and IL-6. Although the causal relations between PD and systemic diseases have not been established, associations have been described between periodontitis and common diseases such as diabetes, pulmonary diseases, osteoporosis, chronic atherosclerosis, stroke, and adverse pregnancy outcomes [19,20]. In fact, there is substantial evidence suggesting that patients with PD have between 1.2–6-fold increased risk of cardiovascular disease, suggesting that inflammation at this mucosal surface has systemic consequences. More strongly, accumulating evidence indicates an association between PD and the development of rheumatoid arthritis (RA) [21]. However, although increasing epidemiological data supports a strong link between PD and RA, both these chronic diseases are multifactorial, and their complex etiologies and pathogenesis themselves remain incompletely understood. However, a recent study by de Aquino et al. [22] demonstrated that concurrent periodontitis induced by repeated oral inoculations of *P*. *gingivalis* and *Prevotella nigrescens* significantly aggravated the severity of collagen-induced arthritis in mice, characterized by increased arthritic bone erosion. Furthermore, the findings from the study provided evidence for the involvement of periodontitis in the pathogenesis of T cell–driven arthritis through induction of a Th17 response. Therefore, in light of these recent findings, it is paramount that future research be directed toward improving our understanding of the links between periodontitis and other chronic inflammatory diseases. This is crucial in terms of raising society's awareness of the connections between oral health and systemic diseases as well as implementation of novel therapeutic strategies for reducing morbidity and mortality of systemic diseases in susceptible individuals.

## Experimental Disease Models and Future Perspectives

Animal models can provide critically important information regarding periodontal disease pathogenesis and have been widely used to establish cause-and-effect relationships and for investigating the efficacy of new treatments. The advantage of using animal models is that they can assess disease progression in a longitudinal manner at many points in time, which is crucial for understanding of the distinct steps in disease initiation or progression. As such, animal models can provide otherwise unattainable information at the host level, despite limitations in their fidelity to all aspects of human periodontal disease development [23]. The two commonly used are the oral gavage model, in which mice are fed large doses of bacteria, and the ligature rat model, in which gingival wounds are induced by tying thread around the tooth surface at the gum level [24]. These rodent models have provided a great deal of information about the process of PD development. The rat ligature model, specifically, has been invaluable in demonstrating that bacteria play an essential role in initiating gingival inflammation and periodontal bone loss, and the oral gavage models have established that certain *A*. *actinomycetemcomitans* and *P*. *gingivalis* virulence factors are essential in promoting an infection [23]. However, no animal model recapitulates all aspects of the disease process, and therefore, more than one model may be needed to provide a better understanding of the various host–pathogen interactions that lead to disease developments.

We now understand that a bacterially induced disruption in host homeostasis is a major factor in the development of PD. However, the microbial etiology remains an enigma as defining the relationship between oral microbial consortia and disease has been precluded by our inability to study complex microbial interactions in the host. The continuous cataloguing of microbial species associated with disease and elucidation of the interspecies interactions in oral biofilm will contribute to our understanding of how these bacteria may act together and result in either health or disease. However, the major challenge facing the study of periodontitis is the development of an animal model incorporating shifts in the oral composition that result in disease. As we begin to gain a greater understanding of the periodontal disease process, it becomes more and more apparent that we need to consider both the bacterial initiator and the host response in a quantitative, time-dependent, site-specific manner if we hope to gain a better understanding of this complex disease.
